# *Drosophila* IAP1-Mediated Ubiquitylation Controls Activation of the Initiator Caspase DRONC Independent of Protein Degradation

**DOI:** 10.1371/journal.pgen.1002261

**Published:** 2011-09-01

**Authors:** Tom V. Lee, Yun Fan, Shiuan Wang, Mayank Srivastava, Meike Broemer, Pascal Meier, Andreas Bergmann

**Affiliations:** 1 Department of Biochemistry and Molecular Biology, Genes and Development Graduate Program, The University of Texas MD Anderson Cancer Center, Houston, Texas, United States of America; 2 Graduate Program in Developmental Biology, Baylor College of Medicine, Houston, Texas, United States of America; 3 The Breakthrough Toby Robins Breast Cancer Research Centre, Institute of Cancer Research, Chester Beatty Laboratories, London, United Kingdom; Stanford University School of Medicine, United States of America

## Abstract

Ubiquitylation targets proteins for proteasome-mediated degradation and plays important roles in many biological processes including apoptosis. However, non-proteolytic functions of ubiquitylation are also known. In *Drosophila*, the inhibitor of apoptosis protein 1 (DIAP1) is known to ubiquitylate the initiator caspase DRONC *in vitro*. Because DRONC protein accumulates in *diap1* mutant cells that are kept alive by caspase inhibition (“undead” cells), it is thought that DIAP1-mediated ubiquitylation causes proteasomal degradation of DRONC, protecting cells from apoptosis. However, contrary to this model, we show here that DIAP1-mediated ubiquitylation does not trigger proteasomal degradation of full-length DRONC, but serves a non-proteolytic function. Our data suggest that DIAP1-mediated ubiquitylation blocks processing and activation of DRONC. Interestingly, while full-length DRONC is not subject to DIAP1-induced degradation, once it is processed and activated it has reduced protein stability. Finally, we show that DRONC protein accumulates in “undead” cells due to increased transcription of *dronc* in these cells. These data refine current models of caspase regulation by IAPs.

## Author Summary

The *Drosophila* inhibitor of apoptosis 1 (DIAP1) readily promotes ubiquitylation of the CASPASE-9–like initiator caspase DRONC *in vitro* and *in vivo*. Because DRONC protein accumulates in *diap1* mutant cells that are kept alive by effector caspase inhibition—producing so-called “undead” cells—it has been proposed that DIAP1-mediated ubiquitylation would target full-length DRONC for proteasomal degradation, ensuring survival of normal cells. However, this has never been tested rigorously *in vivo*. By examining loss and gain of *diap1* function, we show that DIAP1-mediated ubiquitylation does not trigger degradation of full-length DRONC. Our analysis demonstrates that DIAP1-mediated ubiquitylation controls DRONC processing and activation in a non-proteolytic manner. Interestingly, once DRONC is processed and activated, it has reduced protein stability. We also demonstrate that “undead” cells induce transcription of *dronc*, explaining increased protein levels of DRONC in these cells. This study re-defines the mechanism by which IAP-mediated ubiquitylation regulates caspase activity.

## Introduction

Ubiquitylation describes the covalent attachment of ubiquitin, a 76 amino acid polypeptide, to proteins which occurs as a multi-step process (reviewed in [1], [2]). E1-activating enzymes activate ubiquitin and transfer it to E2-conjugating enzymes. E3-ubiquitin ligases mediate the conjugation of ubiquitin from the E2 to the target protein. Repeated ubiquitylation cycles lead to the formation of polyubiquitin chains attached on target proteins. Polyubiquitylated proteins are delivered to the 26S proteasome for degradation. However, non-proteolytic roles of ubiquitylation have also been described (reviewed in [3], [4]). From E1 to E3, there is increasing complexity. For example, the *Drosophila* genome encodes only one E1 enzyme, termed UBA1, which is required for all ubiquitin-dependent reactions in the cell [5]. In contrast, there are hundreds of E3-ubiquitin ligases which are needed to confer substrate specificity.

Programmed cell death or apoptosis is an essential physiological process for normal development and maintenance of tissue homeostasis in both vertebrates and invertebrates (reviewed in [6]). A highly specialized class of proteases, termed caspases, are central components of the apoptotic pathway (reviewed in [7]). The full-length form (zymogen) of caspases is catalytically inactive and consists of a prodomain, a large and a small subunit. Activation of caspases occurs through dimerization and proteolytic cleavage, separating the large and small subunits. Based on the length of the prodomain, caspases are divided into initiator (also known as apical or upstream) and effector (also known as executioner or downstream) caspases [7]. The long prodomains of initiator caspases harbor regulatory motifs such as the caspase activation and recruitment domain (CARD) in CASPASE-9. Through homotypic CARD/CARD interactions with the adapter protein APAF-1, CASPASE-9 is recruited into the apoptosome, a large multi-subunit complex, where it dimerizes and auto-processes leading to its activation [8], [9]. Activated CASPASE-9 cleaves and activates effector caspases (CASPASE-3, -6, and –7), which are characterized by short prodomains. Effector caspases execute the cell death process by cleaving a large number of cellular proteins [10].

In *Drosophila*, the initiator caspase DRONC and the effector caspases DrICE and DCP-1 are essential for apoptosis [11]–[18]. Like human CASPASE-9, DRONC carries a CARD motif in its prodomain [19]. Consistently, DRONC interacts with ARK, the APAF-1 ortholog in *Drosophila* (also known as DARK, HAC-1 or D-APAF-1) [20]–[22] for recruitment into an apoptosome-like complex which is required for DRONC activation [20], [23]–[31]. After recruitment into the ARK apoptosome, DRONC cleaves and activates the effector caspases DrICE and DCP-1 [25], [31]–[34].

Caspases are subject to negative regulation by inhibitor of apoptosis proteins (IAPs) (reviewed in [35], [36]). For example, DRONC is negatively regulated by *Drosophila* IAP1 (DIAP1) [37], [38]. *diap1* mutations cause a dramatic cell death phenotype, in which nearly every mutant cell is apoptotic, suggesting an essential genetic role of *diap1* for cellular survival [39]–[41]. DIAP1 is characterized by two tandem repeats known as the Baculovirus IAP Repeat (BIR), and one C-terminally located RING domain [42]. The BIR domains are required for binding to caspases [37], [38], [43]. The RING domain provides DIAP1 with E3-ubiquitin ligase activity, required for ubiquitylation of target proteins [35], [36]. Importantly, the BIR domains can bind to caspases independently of the RING domain [37], [43].

Usually, IAPs bind to and inhibit activated, i.e. processed caspases, including CASPASE-3, CASPASE-7 and CASPASE-9 as well as the *Drosophila* caspases DrICE and DCP-1 (reviewed in [35], [36]). However, a notable exception to this rule is DRONC. DIAP1 binds to the prodomain of full-length DRONC [37], [38], [43]. This unusual behavior suggests an important mechanism for the control of DRONC activation. Indeed, it has been shown that the RING domain of DIAP1 ubiquitylates full-length DRONC *in vitro* [38], [44]. It has also been proposed that DIAP1 ubiquitylates auto-processed DRONC [33]. These ubiquitylation events are critical for the control of apoptosis, as homozygous *diap1* mutants which lack a functional RING domain (*diap1^ΔRING^*) are highly apoptotic [41]. Because the BIR domains are intact in *diap1^ΔRING^* mutants, binding of DIAP1 to DRONC is not sufficient for inhibition of DRONC under physiological conditions, and ubiquitylation is the critical event for DRONC inhibition.

Although the importance of DIAP1-mediated ubiquitylation of DRONC is well established, it is still unclear how this ubiquitylation event leads to inactivation of DRONC and of caspases in general. Because DRONC protein accumulates in *diap1* mutant cells that are kept alive by expression of the effector caspase inhibitor P35, generating so-called ‘undead’ cells, it has been proposed that DIAP1-mediated ubiquitylation triggers proteasomal degradation of full-length DRONC in living cells, thus protecting them from apoptosis [33], [38], [45], [46]. However, degradation of full-length DRONC in living cells has never been observed and non-degradative models have also been proposed [44]. Furthermore, ubiquitylation of mammalian CASPASE-3 and CASPASE-7 has been demonstrated *in vitro* [47]–[49]. However, evidence for proteasome-dependent degradation of these caspases *in vivo*, i.e. in the context of a living animal, is lacking. In fact, a non-degradative mechanism has been demonstrated for the effector caspase DrICE in *Drosophila* [50].

Here, we further characterize the role of ubiquitylation for the control of DRONC activation. Consistent with a previous report [44], we find that ubiquitylation of DRONC by DIAP1 is critical for inhibition of DRONC's pro-apoptotic activity. Using loss and gain of *diap1* function, we provide genetic evidence that DIAP1-mediated ubiquitylation of full-length DRONC regulates this initiator caspase through a non-degradative mechanism. We find that the conjugation of ubiquitin suppresses DRONC processing and activation. Interestingly, once DRONC is processed and activated, it has reduced protein stability. Finally, we show that *dronc* transcripts accumulate in P35-expressing ‘undead’ cells, accounting for increased DRONC protein levels in these cells. These data refine the current model of caspase regulation by IAPs.

## Results

### Overexpression of DIAP1 fails to suppress apoptosis of *Uba1* mutant cells

**Figure 1:**
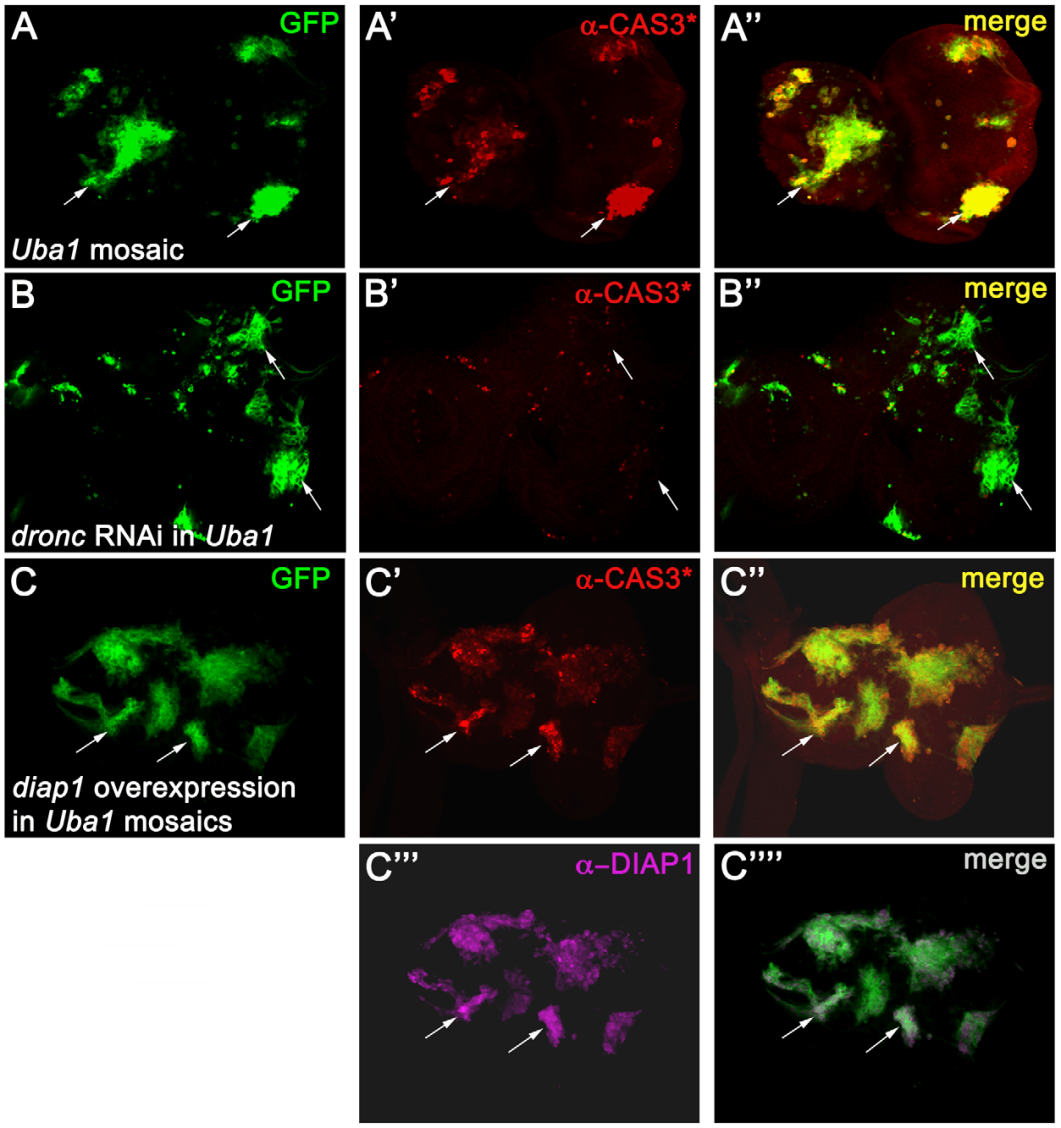
Apoptosis in *Uba1* mutant clones is dependent on DRONC and cannot be inhibited by expression of DIAP1. Shown are eye-antennal imaginal discs from third instar larvae. Posterior is to the right. In each panel, arrows highlight two representative clones. (A) *Uba1* mosaic eye-antennal discs labeled for cleaved CASPASE-3 (α-CAS3*) antibody (red). These discs were incubated at 30°C 12 hours before dissection (see Material and Methods). Presence of GFP marks the location of *Uba1* clones (see arrow). (B) TUNEL labeling of *Uba1* mosaic eye-antennal imaginal discs expressing an RNAi transgene targeting *dronc* (*UAS-droncIR* (inverted repeat)) using the MARCM technique (see Material and Methods). Clones are positively marked by GFP. TUNEL-positive cell death is largely blocked by *dronc* knockdown (B′ and B″). (C) Strong overexpression of *diap1* in *Uba1* clones (magenta in C′″) fails to rescue the apoptotic phenotype, as visualized by CAS3* labeling (red in C′). *Uba1* clones are marked by GFP due to the MARCM technique. Please note that *diap1* is so strongly overexpressed in the clones that we had to adjust the settings in such a way that endogenous DIAP1 in wild-type tissue is below the detection limit (C′″). Genotypes: (A) *hs-FLP UAS-GFP*; *FRT42D Uba1^D6^*/*FRT42D tub*-*Gal80*; *tub*-*GAL4*. (B) *hs-FLP UAS-GFP*; *FRT42D Uba1^D6^*/*FRT42D tub*-*Gal80*; *tub*-*GAL4*/*UAS-droncIR*. (C) *hs-FLP UAS-GFP/UAS-diap1*; *FRT42D Uba1^D6^*/*FRT42D tub*-*Gal80*; *tub*-*GAL4*.

It has previously been shown that complete loss of ubiquitylation due to mutations of the E1 enzyme *Uba1* causes apoptosis in eye imaginal discs as detected by an antibody that recognizes cleaved, i.e. activated, CASPASE-3 (CAS3*) [5], [51], [52] (see also Figure 1A). Because ubiquitylation of DRONC does not occur in *Uba1* mutants, we hypothesized that inappropriate activation of DRONC accounts for the apoptotic phenotype of *Uba1* mutants. To test this possibility, we targeted *dronc* by RNA interference (RNAi) in *Uba1* mutant cells in eye imaginal discs using the MARCM system and labeled for apoptosis using CAS3* antibody. In this system, *Uba1* mutant cells expressing *dronc* RNAi are positively marked by GFP. Consistent with our hypothesis, knock-down of *dronc* strongly reduces apoptosis in *Uba1* mutant clones (Figure 1B). Furthermore, we tested clones doubly mutant for *Uba1* and *ark*, the *Drosophila* ortholog of APAF-1 that is required for DRONC activation (see Introduction). Apoptosis induced in *Uba1* mutant clones is strongly suppressed if *ark* function is removed (Figure S1). These observations suggest that the apoptotic phenotype in *Uba1* clones is caused by inappropriate activation of DRONC, presumably due to lack of ubiquitylation.

However, the protein levels of DIAP1 are increased in *Uba1* mutant clones [5], [52]. There are two possibilities to explain the apoptotic phenotype in *Uba1* mutants despite increased DIAP1 levels. First, the DIAP1 levels may not be sufficiently increased to inhibit DRONC. Alternatively, binding of DIAP1 to DRONC alone may not be sufficient for inhibition of DRONC; instead, ubiquitylation by DIAP1 is required to block DRONC activation, as previously suggested [44]. To distinguish between these two possibilities, we strongly overexpressed *diap1* in *Uba1* mutant clones in eye discs using the MARCM system and imaged for apoptosis by CAS3* labeling. Surprisingly, despite massive expression of *diap1* (>20 fold over wild-type levels; Figure 1C′″), apoptosis still proceeds in *Uba1* mutant clones (Figure 1C′), even though expression of the same transgene can block strong apoptotic phenotypes in several apoptotic paradigms (Figure S2). Apparently, overexpression of DIAP1 is not sufficient to inhibit DRONC and to protect *Uba1* mutant cells from apoptosis. Because DIAP1 is the key regulator of DRONC and because DRONC is required for the apoptotic phenotype of *Uba1* mutant cells, as evidenced by knock-down of *dronc* (Figure 1B), our data provide genetic evidence that binding of DIAP1 is not sufficient for DRONC inhibition in *Uba1* mutant cells.

**Figure 2:**
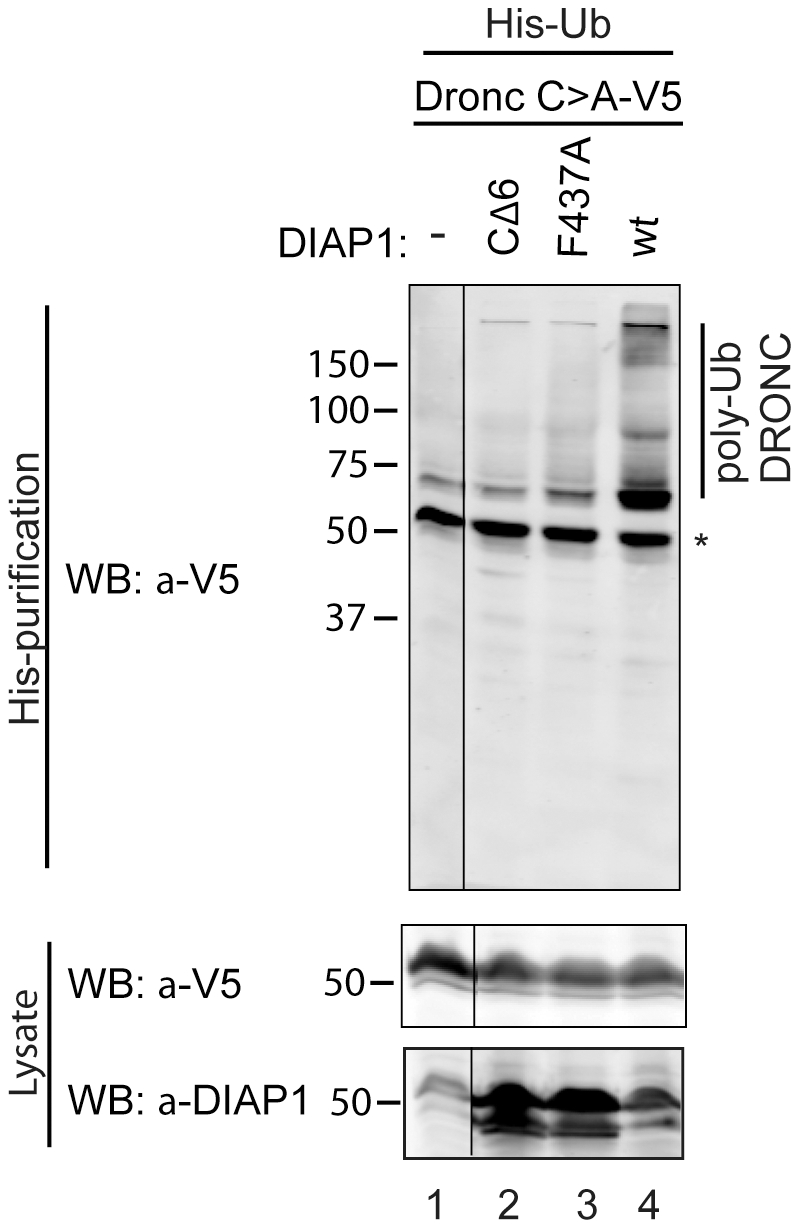
DIAP1 ubiquitylates DRONC in S2 cells. DRONC C>A–V5 was coexpressed with His-Ub and the indicated DIAP1 constructs in S2 cells. Ubiquitylated proteins were purified and analyzed by immunoblot for ubiquitylated DRONC with V5 antibodies. Co-expression of DIAP1^wt^ leads to higher molecular weight modification of DRONC, while the RING-ligase inactive mutants (CΔ6, F437A) cannot ubiquitylate DRONC. * marks non-modified DRONC that is due to unspecific DRONC:matrix association.

Consistent with this view, it has previously been shown that DIAP1 does ubiquitylate full-length DRONC *in vitro* [33], [38], [44]. We tested whether DIAP1 can also ubiquitylate DRONC *in vivo*. Because the available DRONC antibodies failed to immunoprecipitate endogenous DRONC, we transfected DRONC-V5 along with DIAP1^+^ or DIAP1^ΔRING^ mutants (CΔ6, lacking the last six C-terminal residues, and F437A changing a critical Phe residue in the RING domain to Ala [53]) and His-tagged Ubiquitin into *Drosophila* S2 cells. Ubiquitylated proteins were affinity purified under denaturing conditions using Ni columns. The eluates were subsequently examined by immunoblotting with anti-V5 antibodies to detect ubiquitylated forms of DRONC. Under these conditions, DIAP1^+^ readily ubiquitylates full-length DRONC in S2 cells (Figure 2), whereas the RING mutants DIAP1^CΔ6^ and DIAP1^F437A^ were significantly impaired in their ability to ubiquitylate DRONC (Figure 2). These results indicate that DIAP1 ubiquitylates full-length DRONC in a RING-dependent manner in cultured cells.

### Overexpression of DIAP1 does not induce degradation of DRONC

**Figure 3:**
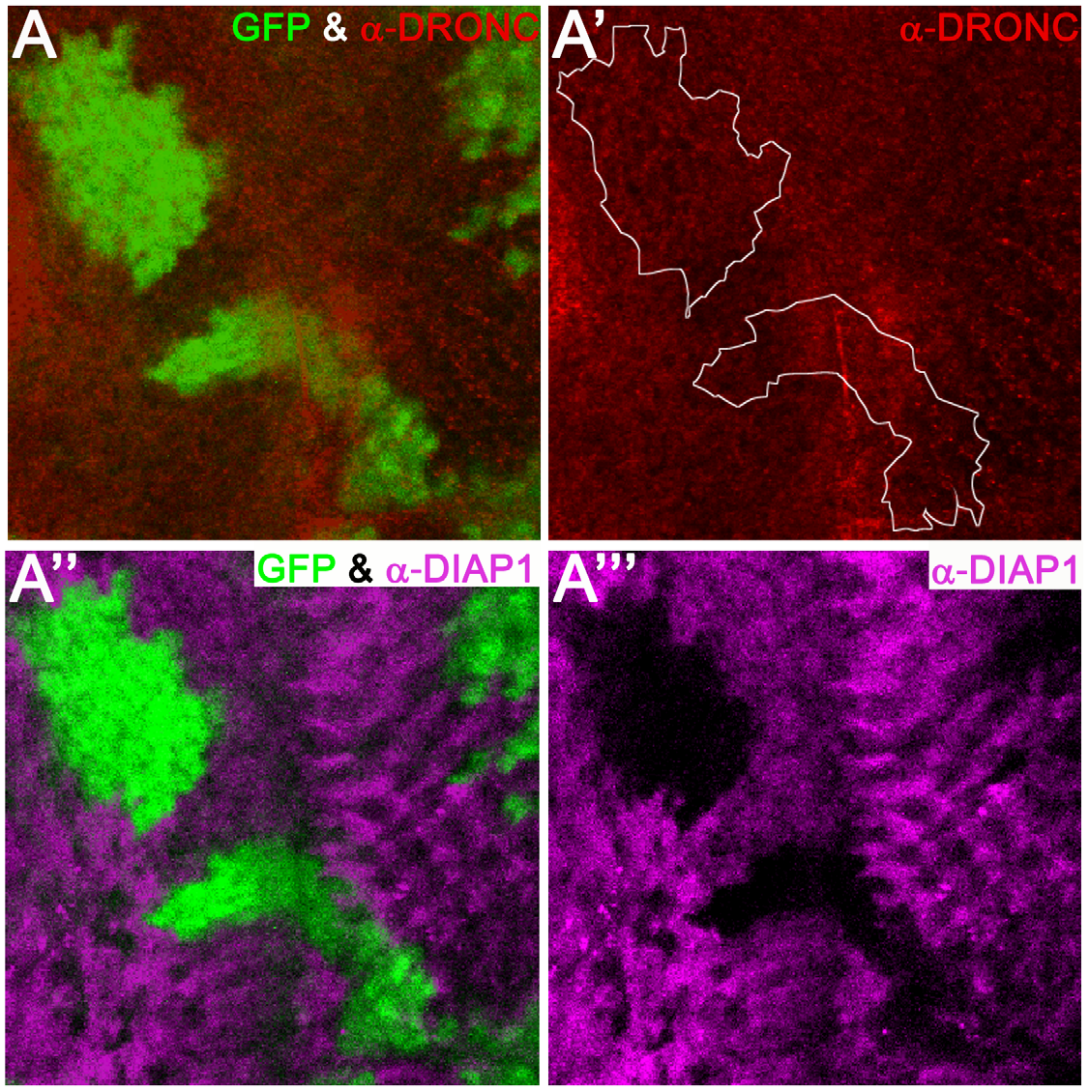
Overexpression of *diap1* does not trigger degradation of DRONC. Shown is an eye imaginal disc from a third instar larva. Posterior is to the right. *diap1*-overexpressing clones are marked by absence of GFP and can be detected using anti-DIAP1 antibodies in magenta (A′″). The boundary between *diap1*-expressing clones and normal tissue is indicated by a white stippled line in (A′). DRONC levels are unchanged (A′). (A) and (A″) are merged images. Genotype: *UAS*-*diap1*/*hs*-*FLP*; *tub*>*GFP*>*GAL4*.

Because DIAP1 readily ubiquitylates DRONC, it has been postulated that DIAP1-mediated ubiquitylation leads to proteasomal degradation of DRONC [33], [38], [45]. However, this has never been rigorously tested *in vivo*. Therefore, we examined, whether overexpression of *diap1* in wild-type animals can influence DRONC protein levels *in vivo*. To this end, we generated clones overexpressing *diap1* (marked by absence of GFP) in eye discs, and analyzed the protein abundance of DRONC. Interestingly, despite high expression of *diap1* (Figure 3A′″), the levels of DRONC remained unchanged and were not influenced by DIAP1 (Figure 3A′). The anti-DRONC antibody used in this assay is specific for DRONC (Figure S3). Importantly, the *diap1* transgene used produces a functional DIAP1 protein that is able to inhibit apoptosis in several paradigms (Figure S2). Therefore, these data suggest that overexpressed DIAP1 does not target DRONC for degradation in living cells.

### REAPER-induced loss of DIAP1 does not increase DRONC protein levels

**Figure 4:**
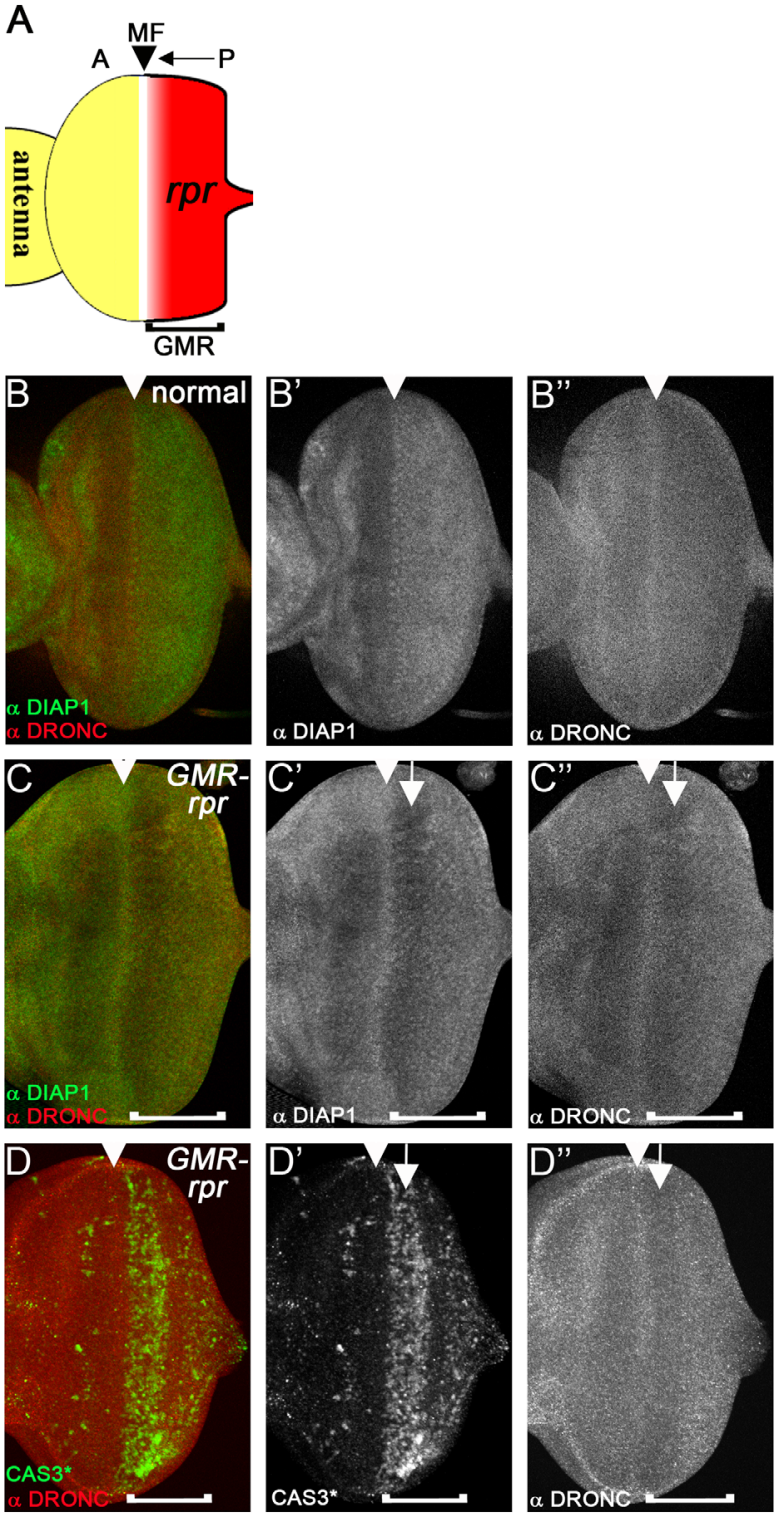
Loss of DIAP1 in *GMR-rpr* eye discs does not alter DRONC protein levels. (A) Schematic illustration of the *GMR*-*reaper* (*GMR*-*rpr*) eye imaginal disc from 3^rd^ instar larvae. The morphogenetic furrow (MF, arrowhead) initiates at the posterior (P) edge of the disc and moves towards the anterior (A) (arrow). The *GMR* enhancer is active posterior to the MF (bracket) and thus expresses *rpr* posterior to the MF (red area). (B-B″) Eye disc showing normal protein distribution of DIAP1 (B′) and DRONC (B″). Both DIAP1 and DRONC levels are increased in the MF (arrowhead). (B) is the merged image of DIAP1 and DRONC labeling. (C–C″) Eye discs expressing two copies of *GMR*-*rpr* eye disc labeled for DIAP1 (C′) and DRONC (C″). Arrowheads mark the MF. DIAP1 levels are markedly reduced posterior to the MF (C′, arrow). Surprisingly, DRONC protein levels are also reduced (C″, arrow). The brackets indicate the extent of *GMR* expression. (D–D″) *2×GMR*-*rpr* eye disc labeled for cleaved CASPASE 3 (CAS3*) (D′) and DRONC (D″). DRONC protein levels are reduced in the CAS3*-positive area (arrow). Arrowheads mark the MF. The brackets indicate the extent of *GMR* expression.

Because of the surprising observation that overexpressed DIAP1 does not cause degradation of DRONC, we tested whether removal of DIAP1 changes DRONC protein levels. Expression of the IAP antagonist *reaper* (*rpr*) induces DIAP1 degradation and apoptosis [54]–[58]. Therefore, we examined whether RPR-induced degradation of DIAP1 changes DRONC protein levels. If DIAP1 targets DRONC for degradation, we would expect that DRONC protein levels would accumulate in response to *rpr* expression. Expression of *rpr* in eye imaginal discs posterior to the morphogenetic furrow (MF) using the *GMR* promoter (*GMR*-*rpr*) is well suited for this analysis. The MF is a dynamic structure that initiates at the posterior edge of the eye disc and moves towards the anterior during 3^rd^ instar larval stage [59], [60] (Figure 4A, arrow). Expression of *rpr* by *GMR* is induced in all cells posterior to the MF [61] (red in Figure 4A). Therefore, *GMR*-*rpr* eye discs provide a continuum of all developmental stages in which cells close to the MF have only recently induced *rpr* expression, while cells towards the posterior edge of the disc have been exposed to *rpr* progressively longer. Therefore, if DRONC accumulates during any of these stages, we should be able to detect it. In wild-type eye discs, DRONC protein is homogenously distributed throughout the disc. Only in the MF, higher levels of DRONC are detectable (arrowhead in Figure 4B″). This high expression of DRONC in the MF serves as an orientation mark. DIAP1 protein levels are low anterior to the MF, but increase in the MF (arrowhead) and posterior to it in wild-type discs (Figure 4B′). In *GMR*-*rpr* eye discs, overall DIAP1 levels are reduced in the *rpr*-expressing domain posterior to the MF (Figure 4C′), but particularly strongly reduced in the CAS3*-positive area (Figure 4C′, D′, arrow) consistent with previous reports [54]–[58]. However, accumulation of DRONC is not observed (Figure 4C″, D″). In contrast, it appears that DRONC levels are also reduced. They are still high in the MF (Figure 4C″, arrowhead), but drop immediately thereafter.

We also related DRONC levels to caspase activation. In the MF, where CAS3* activity is not detectable, DRONC is still high (Figure 4D′, D″; arrowhead), but in the CAS3*-positive area, DRONC levels are reduced (Figure 4D′, D″; arrow). These data indicate that loss of DIAP1 does not cause accumulation of DRONC protein implying that DIAP1 does not induce degradation of DRONC. In contrast, it appears that DIAP1 stabilizes DRONC at least under these conditions (see Discussion).

### “Undead” *diap1* mutant cells induce transcription of *dronc*

**Figure 5:**
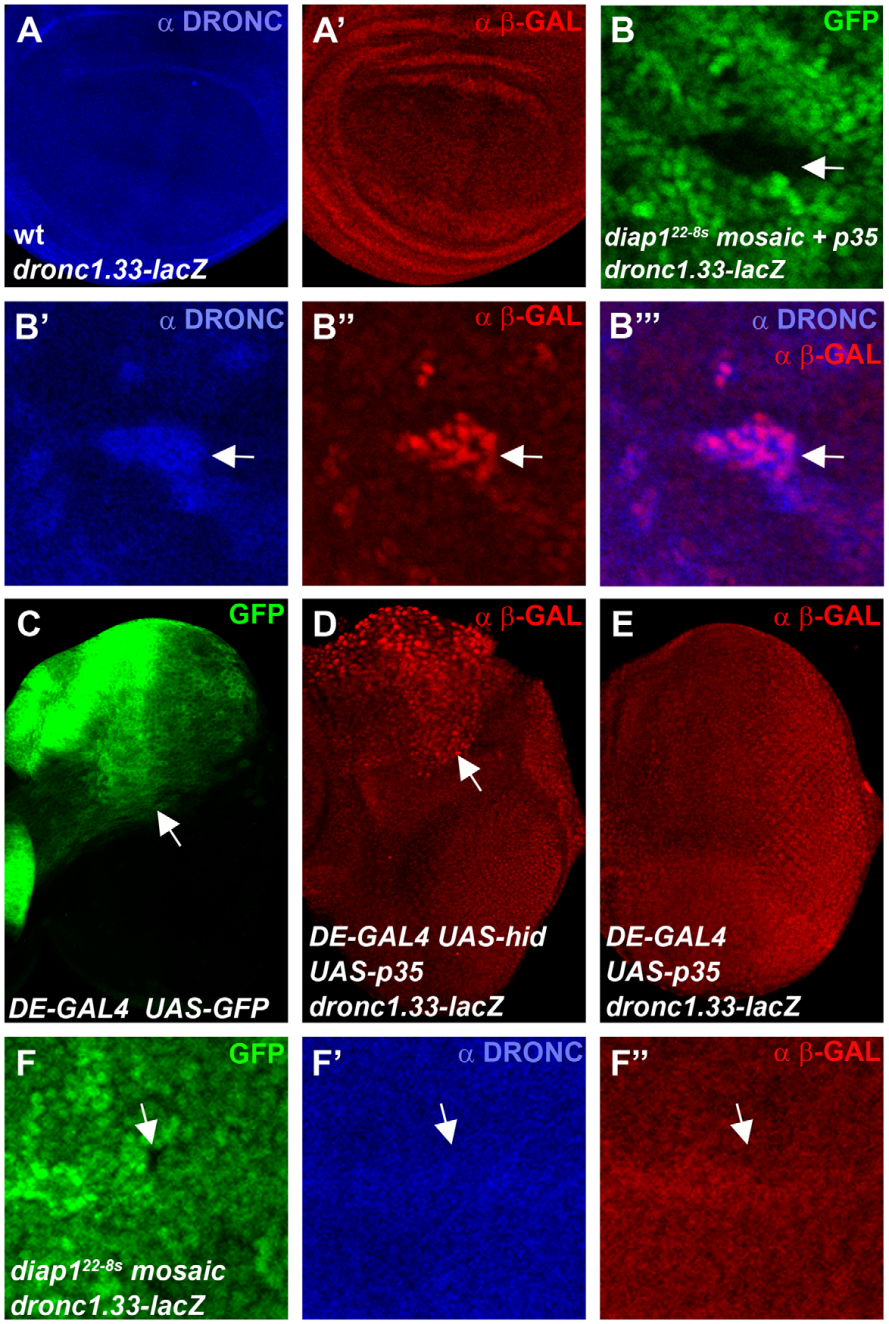
“Undead” *diap1* mutant cells trigger transcription of *dronc*. Shown are 3^rd^ instar larval wing (A,B,F) and eye imaginal discs (C,D,E) labeled for DRONC protein levels (blue) and *dronc* transcriptional activity (red) using the *dronc1.33-lacZ* reporter (ß-GAL labeling). (A,A′) Co-labeling for DRONC protein (A) and *dronc* reporter activity (A′) of a wild-type wing disc expressing the *dronc1.33-lacZ* transgene. (B-B′″) A *diap1^22-8s^* mosaic wing disc expressing *p35* under *nub-GAL4* control in a *dronc1.33-lacZ* background. A mutant clone in the wing pouch is highlighted by an arrow in the GFP-only channel (B). DRONC protein (B′) and ß-GAL immunoreactivity as readout of *dronc1.33-lacZ* activity (B″) are increased in the same cells and overlap (B′″). Please note that the *dronc1.33-lacZ* reporter produces nuclear ß-GAL, while DRONC protein appears cytoplasmic. (C) GFP expression in the eye imaginal disc indicates the dorsal expression domain (arrow) of the *dorsal eye* (*DE*)-*GAL4* driver [95]. (D) Increased *dronc* reporter activity in the dorsal half of the eye imaginal disc (arrow) in undead cells obtained by co-expression of *hid* and *p35* using *DE-GAL4*. (E) Expression of *p35* alone by *DE-GAL4* does not induce *dronc* reporter activity. (F-F″) A *diap1^22-8s^* mosaic wing disc in a *dronc1.33-lacZ* background which does not express *p35*. *diap1^22-8s^* mutant clones are marked by the absence of GFP (F). An arrow points to a representative *diap1^22-8s^* clone in the wing pouch. In the same position, neither DRONC protein (F′) nor *dronc* reporter activity (F″) are increased. Note, that this clone is present in the wing pouch which has the capacity to upregulate DRONC and *dronc* transcription in the ‘undead’, *p35*-expressing condition (see panel B″). Genotypes: (A) *dronc1.33-lacZ*/+. (B) *ubx-FLP*; *nub-GAL4 UAS-p35*/*dronc1.33-lacZ*; *diap1^22-8s^ FRT80*/*ubi-GFP FRT80*. (C) *DE-GAL4 UAS-GFP*/+. (D) *UAS-p35 UAS-hid/dronc1.33-lacZ*; *DE*-*GAL4*. (E) *UAS-p35/dronc1.33-lacZ*; *DE-GAL4*. (F) *ubx-FLP*; *nub*-*GAL4*/*dronc1.33-lacZ*; *diap1^22-8s^ FRT80*/*ubi-GFP FRT80*.

Finally, we analyzed DRONC protein levels in *diap1^ΔRING^* mutants which cannot ubiquitylate DRONC [44]. It has previously been shown that clones of the RING mutant *diap1^22^* ^-*8s*^ accumulate DRONC protein [45], [46] implying that ubiquitylation by the RING domain of DIAP1 causes degradation of DRONC. We repeated these experiments and indeed confirmed that DRONC levels are increased in *diap1^22^* ^-8s^ mutant clones (Figure S4). Thus, these results appear inconsistent with the data presented in Figure 3 and Figure 4 in which manipulating DIAP1 levels did not provide evidence for DIAP1-mediated degradataion of DRONC. However, one caveat with the *diap1^22^* ^-8s^ experiment was the use of the caspase inhibitor P35 which kept *diap1^22^* ^-8s^ mutant cells in an ‘undead’ condition [45]. It has been pointed out that the ‘undead’ state may change the properties of the affected cells (reviewed by [62]) and in fact abnormal induction of transcription in ‘undead’ cells has been reported [45], [63]–[66]. Thus, to explain the conflicting results between the *diap1^22^* ^-8s^ data [45] and our data shown here, we hypothesized that *p35*-expressing ‘undead’ *diap1^22^* ^-*8s*^ clones induce *dronc* transcription, leading to accumulation of DRONC protein. To test this hypothesis, we used a transcriptional *lacZ* reporter containing 1.33 kb of regulatory genomic sequences upstream of the transcriptional start site of the *dronc* gene fused to *lacZ* (*dronc1.33-lacZ*) [67], [68]. Compared to controls (Figure 5A, 5A′) and consistent with the hypothesis, *dronc1.33-lacZ* reporter activity is increased in *p35*-expressing ‘undead’ *diap1^22^* ^-*8s*^ cells in wing imaginal discs and matches the increased DRONC protein pattern (Figure 5B′-5B′″). We also produced ‘undead’ cells in eye imaginal discs by co-expression of the IAP-antagonist *hid* and the caspase inhibitor *p35* in the dorsal half of the eye disc using a *dorsal eye-* (*DE-*) *GAL4* driver (Figure 5C). Similar to wing discs, *dronc* reporter activity is increased in ‘undead’ cells in the dorsal half of the eye (Figure 5D). Expression of *p35* alone does not trigger transcription of *dronc* (Figure 5E) suggesting it is not the mere presence of P35 which causes *dronc* transcription, but the ‘undead’ nature of the affected cells.

These observations may explain why DRONC protein accumulates in ‘undead’ *diap1^22^* ^-*8s*^ mutant cells, but they still do not rule out the possibility that DRONC protein accumulates in *diap1^22^* ^-*8s*^ mutants due to lack of ubiquitylation and thus degradation. To clarify this issue we examined *dronc1.33-lacZ* and DRONC levels in *diap1^22^* ^-*8s*^ mutant clones without simultaneous *p35* expression. Without the inhibition of apoptosis by P35, *diap1^22^* ^-*8s*^ clones die rapidly. Nevertheless, we were able to recover wing discs which contained small *diap1^22-8s^* mutant clones. In these clones, neither *dronc1.33-lacZ* nor DRONC levels are detectably increased (Figure 5F). Notably, these clones are located in the wing pouch in which we observed accumulation of *dronc* reporter activity and DRONC protein under ‘undead’ conditions (Figure 5B″). Thus, the ‘undead’ condition of *p35*-expressing *diap1^22^* ^-*8s*^ mutant cells causes accumulation of DRONC protein due to induction of *dronc* transcription, explaining the observations of Ryoo et al. (2004) [45]. In the absence of *p35* expression, transcription of *dronc* and accumulation of DRONC protein are not observed, providing additional evidence that ubiquitylation of DRONC by the RING domain of DIAP1 does not trigger degradation of DRONC.

### Ubiquitylation controls processing and thus activation of DRONC *in vivo*

**Figure 6:**
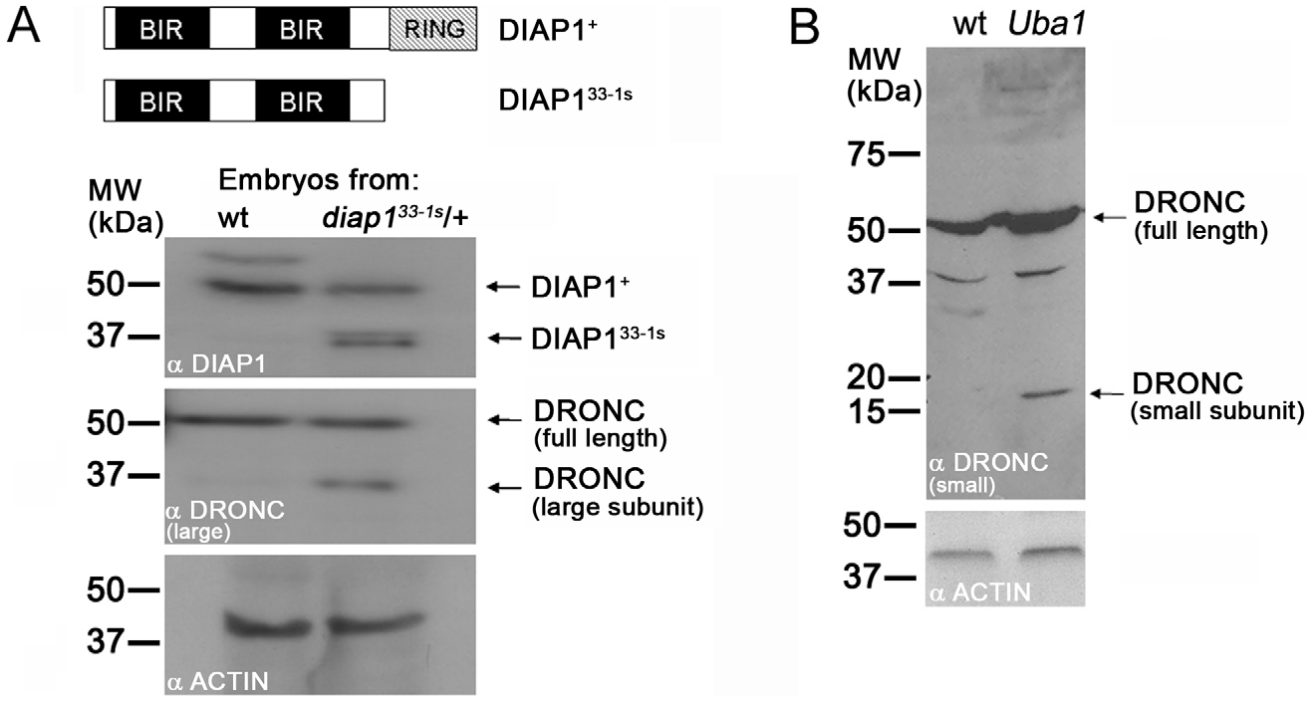
Ubiquitylation controls processing of DRONC. (A) Top: schematic outline of the domain structure of DIAP1^+^ (wild-type) and RING-deleted DIAP1^33-1s^. Not drawn to scale. Immunoblots of embryonic extracts of stage 6–9 wild-type (wt) and heterozygous *diap1^33-1s^* mutants were probed with anti-DIAP1 (upper panel) and anti-DRONC antibodies (middle panel). The RING-depleted *diap1^33-1s^* allele produces a stable protein (DIAP1^33-1s^) that is detectable by its faster electrophoretic mobility (upper panel). In RING-depleted *diap1^33-1s^* embryos a significant portion of processed DRONC is present (middle panel) which likely accounts for the apoptotic phenotype of *diap1^33-1s^* embryos [41]. These extracts were obtained from a cross of heterozygous males and females. Thus, only one quarter of the embryos is homozygous mutant for *diap1^33-1s^*; yet, processed DRONC is easily detectable. The anti-DRONC antibody is specific for the large subunit of DRONC. Lower panel: loading control. (B) Extracts of imaginal discs from wild-type (wt) and mosaic *Uba1* imaginal discs were analyzed by immunoblotting using an antibody raised against the small subunit of DRONC. Clones of the temperature sensitive allele *Uba1^D6^* were induced at the permissive temperature in first larval instar and then shifted to the non-permissive temperature (30°C) during third larval instar 12 hours before dissection (see Material and Methods). This treatment ensures that approximately 50% of the mosaic disc is mutant for *Uba1*. Although only 50% of the disc tissue is mutant for *Uba1*, processed DRONC is easily detectable. Lower panel: loading control.

Our *in vivo* analysis implies that DIAP1-mediated ubiquitylation does not trigger proteasomal degradation of DRONC. To identify the role of ubiquitylation for regulation of DRONC activity, we analyzed the fate of DRONC protein in RING mutants of *diap1*. Of note, these mutants retain their ability to bind to DRONC, because DRONC binding is not mediated by the RING domain, but by the BIR2 domain [37], [38], [43]. The RING mutant *diap1^33-1s^* is especially suitable for this analysis because a premature stop codon results in deletion of the entire RING domain but leaves the BIR domains intact [44] (Figure 6A), thus abrogating its E3 activity, but not caspase binding. Importantly, *diap1^33-1s^* is characterized by a strong apoptotic phenotype, suggesting inappropriate caspase activation [41], [45]. Indeed, we showed previously that *diap1^ΔRING^* mutant phenotypes are dependent upon DRONC, because *dronc* mutants suppress *diap1^ΔRING^* phenotypes [11]. Therefore, ubiquitylation of DRONC by DIAP1 is critical to maintain cell survival.

We examined the cause of the *diap1^33^* ^-*1s*^ apoptotic phenotype. First, as a control, we determined whether the *diap1^33^* ^-*1s*^ gene produces a stable protein *in vivo*. We chose to analyze stage 6–9 embryos, because normal developmental cell death starts at stage 11 [69]; therefore, stage 6–9 *diap1^33^* ^-*1s*^ mutant embryos allow analysis of DIAP1 in the absence of upstream apoptotic signals. In immunoblots of embryonic extracts obtained from a cross of heterozygous *diap1^33^* ^-*1s*^ males and females, the DIAP1^33-1s^ protein is easily distinguished from wild-type DIAP1^+^ protein due to its faster electrophoretic mobility (Figure 6A, top panel). The presence of the DIAP1^33-1s^ protein suggests that the apoptotic phenotype in *diap1^33^* ^-*1s*^ mutant embryos is not caused by instability of the mutant protein. Interestingly, the protein levels of DIAP1^+^ and RING-deleted DIAP1^33-1s^ are similar (Figure 6A, top panel) suggesting that loss of the RING domain does not influence the protein stability of DIAP1 in the absence of pro-apoptotic signals.

Next, we analyzed DRONC protein in extracts from *diap1^33^* ^-*1s*^ mutant embryos. Consistent with the data in Figure 4 and Figure 5, we do not detect a significant increase in the protein levels of DRONC in these embryos (Figure 6A, middle panel). However, a significant amount of DRONC is present in a processed form in extracts of stage 6–9 *diap1^33-1s^* mutant embryos which is absent in control extracts from wild-type embryos (Figure 6A, middle panel). Therefore, DRONC processing, and thus activation, occurs in RING-depleted *diap1^33^* ^-*1s*^ mutant embryos despite the fact that the BIR domains of DIAP1 are unaffected. The processed form of DRONC in this mutant of MW ∼36 kDa corresponds to the major apoptotic form of DRONC composed of the large subunit and the prodomain of DRONC [70]. This finding, and the one presented in Figure 1, confirms that binding of DIAP1 to DRONC is not sufficient to prevent processing and activation of DRONC. Instead, the RING domain is required to control DRONC processing. Because the RING domain contains an E3-ubiquitin ligase activity [45], [55]–[58] and because ubiquitylation of full-length DRONC does not trigger proteasomal degradation (Figure 3, Figure 4, and Figure 5), we conclude that ubiquitylation of DRONC by the RING domain of DIAP1 is necessary to inhibit DRONC processing and thus activation.

To further characterize the role of ubiquitylation in the regulation of DRONC processing, we performed an immunoblot analysis with extracts from wild-type and *Uba1* mosaic imaginal discs, which, under our experimental conditions, are about half mutant for *Uba1* and half wild-type. Immunoblot analysis demonstrated that a significant amount of DRONC is processed in *Uba1* mosaic discs (Figure 6B). Thus, these data further support the notion that ubiquitylation of full-length DRONC is necessary for inhibition of DRONC processing.

## Discussion

In this paper, we provide three take-home messages. First, we provide genetic evidence that binding of DIAP1 to DRONC is not sufficient for inhibition of DRONC. Instead, ubiquitylation of DRONC controls its apoptotic activity, consistent with the apoptotic phenotype of *diap1^ΔRING^* mutants, that retain caspase binding abilities. Second, DIAP1-mediated ubiquitylation of full-length DRONC does not lead to its proteasomal degradation; rather, ubiquitylation directly controls processing and activation of DRONC. Interestingly, processed and active DRONC shows reduced protein stability. Third, ‘undead’ cells accumulate *dronc* transcripts.

### Binding of DIAP1 is not sufficient for Dronc inhibition

Based on biochemical studies *in vitro* and overexpression studies in cultured cells, many of cancerous origin, it was initially proposed that binding of IAPs to caspases through their BIR domains is sufficient to inhibit caspases [71]–[80]. However, when ubiquitylation of caspases by IAPs was described [38], [44], [47], [48], it was unclear what role ubiquitylation would play for control of caspase activity, especially since for none of them, ubiquitin-mediated degradation has been observed (see below). Because the RING domain is also required for auto-ubiquitylation of DIAP1 [54]–[58], mutations of the RING domain would be expected to increase DIAP1 protein levels and protect cells from apoptosis. However, the opposite phenotype, massive apoptosis, was observed [41]. Nevertheless, despite the biochemical studies showing that the BIR domains of DIAP1 are sufficient for interaction with DRONC [37], [38], [43], one could argue that DIAP1^ΔRING^ mutants have lost the ability to interact with DRONC *in vivo*. While we cannot exclude this possibility due to the inability of our antibodies to immunoprecipitate endogenous proteins, another experiment supports the notion that ubiquitylation is necessary for DRONC inhibition: when wild-type *diap1* was strongly overexpressed in an ubiquitylation-deficient *Uba1* mutant background, DRONC-dependent apoptosis was not inhibited (Figure 1C), suggesting that binding of DIAP1 is not sufficient for inhibition of DRONC. Instead, ubiquitylation is critical for inhibition of DRONC activity.

### DIAP1 does not control protein levels of full-length DRONC

The current model holds that DIAP1-mediated ubiquitylation leads to proteasomal degradation of full-length DRONC in living cells [33], [38], [45]. However, our data do not support this model *in vivo*. This model is based on biochemical ubiquitylation studies without *in vivo* validation and does not take into account that ubiquitylation often serves non-proteolytic functions [1], [3], [4]. Both overexpression and loss of *diap1* does not cause a detectable alteration of the protein levels of DRONC (Figure 3, Figure 4, Figure 5), arguing against a degradation model. The only example where DRONC accumulation has been observed is in P35-expressing ‘undead’ *diap1^ΔRING^* mutant cells [45], [46], and we showed here that the ‘undead’ nature of these cells causes transcriptional induction of *dronc* (Figure 5). Together, these observations argue against a degradation model of full-length DRONC and favor a non-traditional (non-proteolytic) role of ubiquitylation regarding control of DRONC activity. Similarly, DIAP1-mediated ubiquitylation of the effector caspase DrICE inactivates this effector caspase through a non-degradative mechanism [50].

Interestingly, in *GMR-rpr* eye imaginal discs, DRONC protein levels appear to be reduced in apoptotic cells compared to living cells (Figure 4C″, 4D″). Due to the apoptotic activity of REAPER, DRONC is present in its processed and activated form. Reduced protein stability of DRONC has also been reported after incorporation into the ARK apoptosome where it is processed and activated [46]. Combined, these observations suggest that while DIAP1-mediated ubiquitylation of full-length DRONC does not trigger its degradation, processed and activated DRONC has reduced protein stability and may indeed be degraded. It is unclear whether degradation of activated DRONC is mediated by DIAP1, as proposed previously [33]. In *GMR-rpr* eye imaginal discs, reduced DRONC levels correlate with a reduction of DIAP1 protein (Figure 4C′, 4D′). This correlation indicates that DIAP1 may actually stabilize DRONC protein, at least in part. Alternatively, because DRONC and DIAP1 form a complex [37], REAPER-induced degradation of DIAP1 may result in co-degradation of complexed DRONC in the process. Further studies are needed to determine the cause of decreased DRONC stability in apoptotic cells.

In addition to *Drosophila* DRONC, mammalian CASPASE-3 and CASPASE-7 have been reported to be ubiquitylated *in vitro* [47], [48]. However, proteasome-mediated degradation of these caspases *in vivo* has not been reported. Although a decrease of CASPASE-3 levels has been noted upon overexpression of XIAP, this was shown for an artificial CASPASE-3 mutant, in which the order of the subunits was reversed and the Cys residue in the active site changed to Ser [47]. This catalytically inactive CASPASE-3 mutant is not proteolytically processed [47]. Therefore, physiological *in vivo* evidence for IAP-mediated degradation of mammalian caspases is lacking.

Moreover, the phenotype of a RING-deleted *XIAP* mutant mouse is consistent with our data [49]. The *XIAP^ΔRING^* mutant, which was generated by a knock-in approach in the endogenous *XIAP* gene, is characterized by increased caspase activity [49]. Intriguingly, the protein levels of CASPASE-3, CASPASE-7 and CASPASE-9 did not significantly change in the *XIAP^ΔRING^* mutant despite the fact that ubiquitylation of CASPASE-3 was reduced. However, processing of these caspases was easily detectable in *XIAP^ΔRING^* mutants [49]. These data are very similar to the ones presented here for *diap1^33^* ^-1s^ (Figure 6), and together strongly suggest that non-proteolytic ubiquitylation controls caspase processing and activity in both vertebrates and invertebrates.

Non-proteolytic roles of ubiquitylation have been described in recent years and are involved in many processes including signal transduction, endocytosis, DNA repair, and histone activity (reviewed in [1], [3], [4]). Two types of ubiquitylation lead to non-proteolytic functions. Monoubiquitylation is involved in endocytosis, DNA repair and histone activity. In fact, mammalian CASPASE-3 and CASPASE-7 have been found to be monoubiquitylated *in vitro* [48]. However, it is unclear whether DRONC is monoubiquitylated by DIAP1. The presence of high molecular-weight ubiquitin conjugates *in vitro* (Figure 2) suggests that DRONC may be polyubiquitylated, at least under the experimental conditions [38], [44].

Polyubiquitylation serves both proteolytic and non-proteolytic functions depending on the Lysine (K) residue used for polyubiquitin chain formation. In general, if polyubiquitylation occurs via K48, the target protein is subject to proteasome-mediated degradation. If it occurs on a different Lys residue, such as K63, non-proteolytic functions are induced [1], [3], [4]. The best studied examples of both K48- and K63-linked polyubiquitylation are in the NF-κB pathway (reviewed in [3], [81]). While K48-polyubiquitylation leads to proteasomal degradation, K63-linked polyubiquitin chains act as scaffolds to assemble protein complexes required for NF-κB activation [3], [81]. It is unknown what type of polyubiquitin chain is attached to DRONC, but it is possible that it is not K48-linked. Interestingly, in this context it has been shown that auto-ubiquitylation of DIAP1 preferentially involves K63-linked polyubiquitin chains [82]. By analogy, DIAP1 may ubiquitylate DRONC through formation of K63-linked polyubiquitin chains. This will be an interesting avenue to explore in future experiments.

Conjugated monoubiquitin and polyubiquitin chains can serve as docking sites for factors containing ubiquitin-binding domains (UBD) [2], [4], [83]. The UBD-containing factors interpret the ubiquitylation status of the target protein, and trigger the appropriate response. For example, K48-linked polyubiquitin chains are recognized by Rad23 and Drk2 which deliver them to the proteasome [2]. Other forms of ubiquitin conjugates are recognized by different UBD-containing factors which control the activity of the ubiquitylated protein. Therefore, it is possible that an as yet unknown UBD-containing protein binds to ubiquitylated DRONC and controls its activity. For example, such an interaction could block the recruitment of ubiquitylated DRONC into the ARK apoptosome. Another possibility is that ubiquitylation could block dimerization of DRONC, which is required for activation of DRONC [34].

### “Undead” cells trigger *dronc* transcription

‘Undead’ cells can be obtained by expression of the effector caspase inhibitor P35 [84]. In these cells, apoptosis has been induced, but cannot be executed due to effector caspase inhibition. Nevertheless, the initiator caspase DRONC is active in ‘undead’ cells and can promote non-apoptotic processes [51]. Recent work has suggested that ‘undead’ cells may alter their cellular behavior. For example, ‘undead’ cells change their size and shape, and have some migratory abilities to invade neighboring tissue [62]. They are also able to promote proliferation of neighboring cells causing hyperplastic overgrowth [15], [45], [63]–[66] (reviewed by [85], [86]). Altered transcription of the TGF-ß/BMP member *decapentaplegic* (*dpp*), the Wnt-homolog *wingless* (*wg*), and the p53 ortholog *dp53* has also been reported in ‘undead’ cells [45], [64]–[66]. Intriguingly, while *dpp* and *wg* are usually not expressed in the same cells [87], ‘undead’ cells co-express them ectopically, clearly indicating an altered transcriptional program.

As part of this altered transcriptional program, we showed that ‘undead’ cells also stimulate transcription of the initiator caspase *dronc* (Figure 5). Interestingly, *p35* expression in normal cells does not induce *dronc* transcription suggesting that it is not the mere presence of P35 that induces *dronc* transcription, but instead the ‘undead’ condition of the affected cells. This transcriptional induction of *dronc* provides an explanation why DRONC protein levels are increased in ‘undead’ cells. It may also help to explain another observation regarding ‘undead’ cells. It has been demonstrated that although these cells are unable to die, they maintain the apoptotic machinery indefinitely [62], [88]. Therefore, as part of this maintenance program, ‘undead’ cells stimulate *dronc* transcription. This is likely not restricted for *dronc*. Martin et al. (2009) [62] also showed that DrICE protein levels remain high in ‘undead’ cells which may also be caused by increased *drICE* transcription. It is also possible that the induction of *dp53* by ‘undead’ cells [66] is part of this maintenance program, because we have shown that Dp53 induces expression of *hid* and *rpr* [89] and a positive feedback loop between *dp53*, *hid* and *dronc* exists in ‘undead’ cells [66]. This may all occur at a transcriptional level. The mechanism by which ‘undead’ cells stimulate expression of *dpp*, *wg*, *dp53*, *dronc* and potentially *drICE* are currently unknown and are avenues for future research.

## Material and Methods

### *Drosophila* genetics

Fly crosses were conducted using standard procedures at 25°C. The following mutants and transgenes were used: *Uba1^D6^* [5]; *ark^G8^* [26]; *diap1^22-8s^* and *diap1^33^* ^-1s^ [44]; *vps25^N55^* [90]; *dronc^I29^* [11]; *UAS-droncIR* (*dronc* inverted repeats) [91]; *GMR-rpr* [92]; *dronc1.33-lacZ* [67], [68], *ubx-FLP* [93], *nub-GAL4* [94], *DE-* (*dorsal eye-*) *GAL4* [95], and *UAS-hid* [96]. *nub-FLP* is *nub-GAL4 UAS-FLP*. *UAS-p35* and *UAS-FLP* were obtained from Bloomington. *Uba1^D6^* is a temperature sensitive allele which at 25°C is a hypomorphic allele, but at 30°C is a null allele [5]. In the experiments in Figure 1, Figure 6B, and Figure S1, *Uba1^D6^* and *Uba1^D6^ ark^G8^* mosaic larvae were incubated at 25°C; 12 hours before dissection the temperature was shifted to 30°C. This treatment allows recovery of *Uba1^D6^* null mutant clones, which otherwise are cell lethal.

### Generation of mutant clones and expression of transgenes

Mutant clones were induced in eye-antennal imaginal discs using the *FLP*/*FRT* mitotic recombination system [97] using *ey*-*FLP* [98]. In this case, clones are marked by loss of GFP. Expression of *diap1* and *dronc* RNAi in *Uba1^D6^* clones (Figure 1) was induced from *UAS-diap1* or *UAS-droncIR* transgenes using the MARCM system [99]. Here, clones are positively marked by GFP. For induction of *diap1*-expressing clones in Figure 3, the *UAS-diap1* transgene was crossed to *hs*-*FLP*; *tub*<*GFP*<*GAL4* (< = FRT). Clones are marked by the absence of GFP. MARCM clones and *diap1*-overexpressing clones were induced in first instar larvae by heat-shock for one hour in a 37°C water bath. Expression of *UAS-p35* in *diap1^22-8s^* mosaic discs was accomplished by *nub*-*GAL4*.

### Immunohistochemistry

Eye-antennal imaginal discs from third instar larvae were dissected using standard protocols and labeled with antibodies raised against the following antigens: DIAP1 (a kind gift of Hermann Steller and Hyung Don Ryoo); cleaved CASPASE-3 (CAS3*) (Cell Signaling Technology) and anti-ß-GAL (Promega). The DRONC antibody used for immunofluorescence was raised against the C-terminus of DRONC in guinea pigs [44]. This antibody is specific for DRONC (Figure S3). Cy3- and Cy-5 fluorescently-conjugated secondary antibodies were obtained from Jackson ImmunoResearch. In each experiment, multiple clones in 10–20 eye and wing imaginal discs were analyzed, unless otherwise noted. Images were captured using an Olympus Optical FV500 confocal microscope.

### Ubiquitylation assays

Schneider S2 cells were co-transfected with pMT-DRONC C>A V5, pAcDIAP1 (wt or CΔ6, F437A, respectively, described in [50]) and pAc His-HA-Ub at equal ratios. Expression of DRONC was induced overnight with 350 µM CuSO_4_. Cells were lysed under denaturing conditions and ubiquitylated proteins were purified using Ni^2+^-NTA agarose beads (QIAGEN). Immunoblot analysis was performed with α-V5 (Serotec) and α-DIAP1 antibodies [37], [43].

### Immunoblot analysis

For the immunoblots in Figure 6A, embryos were collected, decorionated and snap frozen in liquid nitrogen. Embryos were sonicated in Laemmli SDS loading buffer while being frozen. The equivalent of 30 lysed embryos was loaded per lane. Immunoblots were done using standard procedures. The anti-DRONC antibody used in Figure 6A is a peptide antibody raised against the large subunit of DRONC. The anti-DRONC antibody used in Figure 6B was raised against the C-terminus of DRONC in guinea pigs.

## Supporting Information

Figure S1 Loss of *ark* suppresses apoptosis in *Uba1* clones. *Uba1 ark* mosaic eye-antennal disc labeled for cleaved CASPASE-3 (CAS3*) antibody (red). These discs were incubated at 30°C 12 hours before dissection (see Material and Methods). Absence of GFP marks the location of *Uba1 ark* clones (see arrows). There is scattered apoptosis detectable. However, this occurs throughout the disc and does not correlate with the positions of the *Uba1 ark* double mutant clones. Genotype: *ey-FLP*; *FRT42D Uba1^D6^ ark^G8^*/*FRT42D ubi-GFP*.
(TIF)

Figure S2 *UAS-diap1* rescues *GMR-hid* and apoptosis induced in *vps25* mutants. Because the *UAS-diap1* transgene failed to suppress apoptosis in *Uba1* clones (Figure 1C), we tested its ability to inhibit the strong apoptotic phenotype in two other paradigms. (A) Overexpression of the IAP-antagonist *hid* specifically in the fly eye under *GMR* promoter control gives rise to a strong eye ablation phenotype due to massive induction of apoptosis [100]. (B) Coexpression of *UAS-diap1* partially suppresses the *GMR-hid* eye ablation phenotype [42]. (C) *vps25* mutant clones induce a strong apoptotic phenotype. *vps25* encodes an component involved in endosomal protein sorting [90]. The apoptotic phenotype of *vps25* and *Uba1* as well as other phenotypes caused by inactivation of these genes are very similar, and both mutants were obtained in the same genetic screen [5], [90]. The left panel is the merge of GFP and anti-cleaved CASPASE-3 (CAS3*) labeling, the right panel (C′) displays only the CAS3* channel. White arrows mark a few clones as examples. (D) Overexpression of *diap1* completely suppresses the strong apoptotic phenotype of *vps25* mutant clones. The experimental conditions applied here are identical to the *Uba1* experiment in Figure 1C. The left panel is the merge of GFP and anti-cleaved CASPASE-3 (CAS3*) labeling, the right panel (D′) displays only the CAS3* channel. Genotype: *hs-FLP UAS-GFP/UAS-diap1*; *FRT42D vps25^N55^*/*FRT42D tub*-*Gal80*; *tub*-*GAL4*. Genotypes: (A) *GMR-hid GMR-GAL4*. (B) *UAS-diap1; GMR-hid GMR-GAL4*. (C) *ey-FLP*; *FRT42D vps25^N55^*/*FRT42D* P[*ubi-GFP*]. (D) *ey-FLP*; *FRT42D vps25^N55^*/*FRT42D* P[*ubi-GFP*].
(TIF)

Figure S3 Specificity of the anti-DRONC antibody. The specificity of the anti-DRONC antibody used for immunofluorescence in Figure 3, Figure 4, and Figure 5 was verified in *dronc^I29^* mosaic eye (A) and wing (B) imaginal discs. The *dronc^I29^* allele contains a premature STOP codon at position 53 [11]. *dronc^I29^* clones were induced using the MARCM system, hence they are positively marked by GFP (arrows). The anti-DRONC antibody does not produce labeling signals in the mutant clones (arrows in A′ and B′, and the merge in A″ and B″), demonstrating that it is specific for DRONC. Genotype: *hs*-*FLP*; *dronc^I29^ FRT80*/*ubi*-*GFP FRT80*.
(TIF)

Figure S4 “Undead” *diap1^22-8s^* cells accumulate DRONC protein autonomously. (A, A′) Using MARCM, *p35*-expressing, ‘undead’ *diap1^22-8s^* mutant clones (green) were induced in eye discs and labeled for DRONC protein (red). DRONC protein autonomously accumulates in P35-expressing *diap1^22-8s^* clones (arrows). Similar results were obtained in wing discs (data not shown). Genotype: *hs-FLP tub-GAL4 UAS-GFP*/+; *UAS-p35*/+; *diap1^22-8s^ FRT80*/*tub-GAL80 FRT80.*
(TIF)
