# Supplementary figures and images for: Drosophila IAP1-Mediated Ubiquitylation Controls Activation of the Initiator Caspase DRONC Independent of Protein Degradation
Source: PLoS Genet. 2011 Sep 1;7(9):e1002261. doi: 10.1371/journal.pgen.1002261 (PMC3164697; doi:10.1371/journal.pgen.1002261)

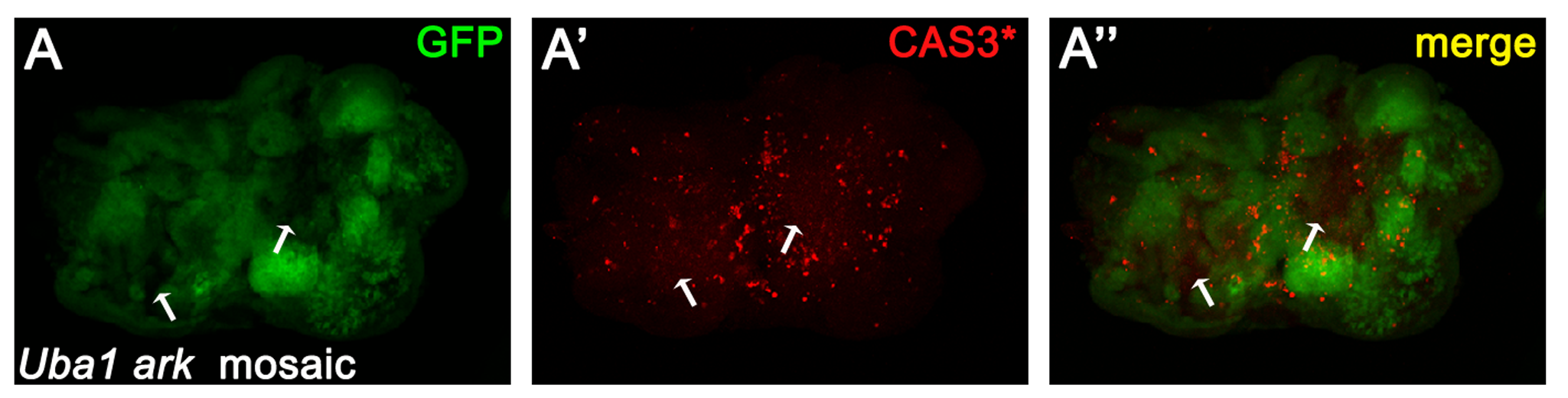

Supplement: Figure S1 — Loss of ark suppresses apoptosis in Uba1 clones. Uba1 ark mosaic eye-antennal disc labeled for cleaved CASPASE-3 (CAS3*) antibody (red). These discs were incubated at 30°C 12 hours before dissection (see Material and Methods). Absence of GFP marks the location of Uba1 ark clones (see arrows). There is scattered apoptosis detectable. However, this occurs throughout the disc and does not correlate with the positions of the Uba1 ark double mutant clones. Genotype: ey-FLP; FRT42D Uba1D6 arkG8/FRT42D ubi-GFP. (TIF) [file pgen.1002261.s001.tif]

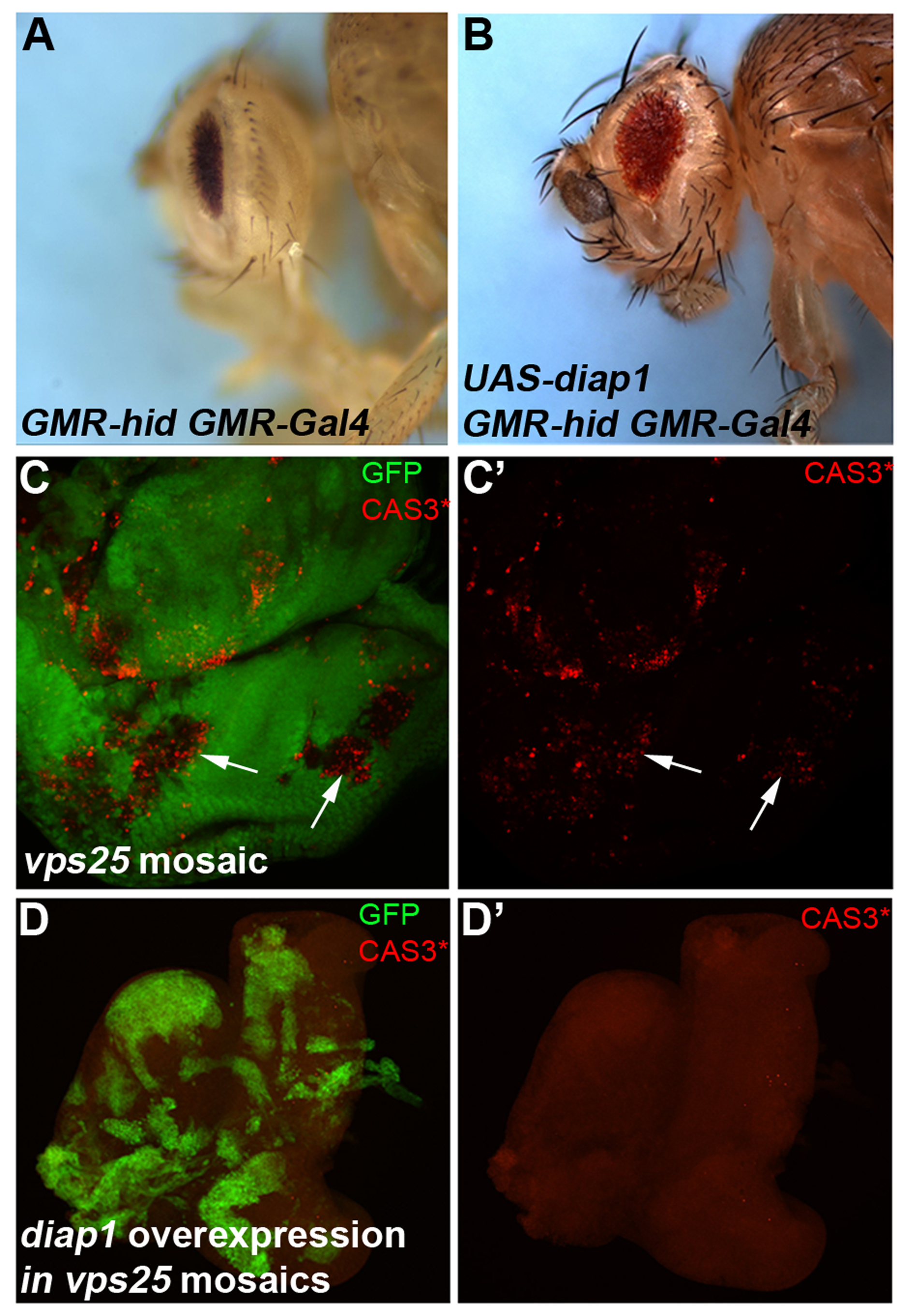

Supplement: Figure S2 — UAS-diap1 rescues GMR-hid and apoptosis induced in vps25 mutants. Because the UAS-diap1 transgene failed to suppress apoptosis in Uba1 clones (Figure 1C), we tested its ability to inhibit the strong apoptotic phenotype in two other paradigms. (A) Overexpression of the IAP-antagonist hid specifically in the fly eye under GMR promoter control gives rise to a strong eye ablation phenotype due to massive induction of apoptosis [100]. (B) Coexpression of UAS-diap1 partially suppresses the GMR-hid eye ablation phenotype [42]. (C) vps25 mutant clones induce a strong apoptotic phenotype. vps25 encodes an component involved in endosomal protein sorting [90]. The apoptotic phenotype of vps25 and Uba1 as well as other phenotypes caused by inactivation of these genes are very similar, and both mutants were obtained in the same genetic screen [5], [90]. The left panel is the merge of GFP and anti-cleaved CASPASE-3 (CAS3*) labeling, the right panel (C′) displays only the CAS3* channel. White arrows mark a few clones as examples. (D) Overexpression of diap1 completely suppresses the strong apoptotic phenotype of vps25 mutant clones. The experimental conditions applied here are identical to the Uba1 experiment in Figure 1C. The left panel is the merge of GFP and anti-cleaved CASPASE-3 (CAS3*) labeling, the right panel (D′) displays only the CAS3* channel. Genotype: hs-FLP UAS-GFP/UAS-diap1; FRT42D vps25N55/FRT42D tub-Gal80; tub-GAL4. Genotypes: (A) GMR-hid GMR-GAL4. (B) UAS-diap1; GMR-hid GMR-GAL4. (C) ey-FLP; FRT42D vps25N55/FRT42D P[ubi-GFP]. (D) ey-FLP; FRT42D vps25N55/FRT42D P[ubi-GFP]. (TIF) [file pgen.1002261.s002.tif]

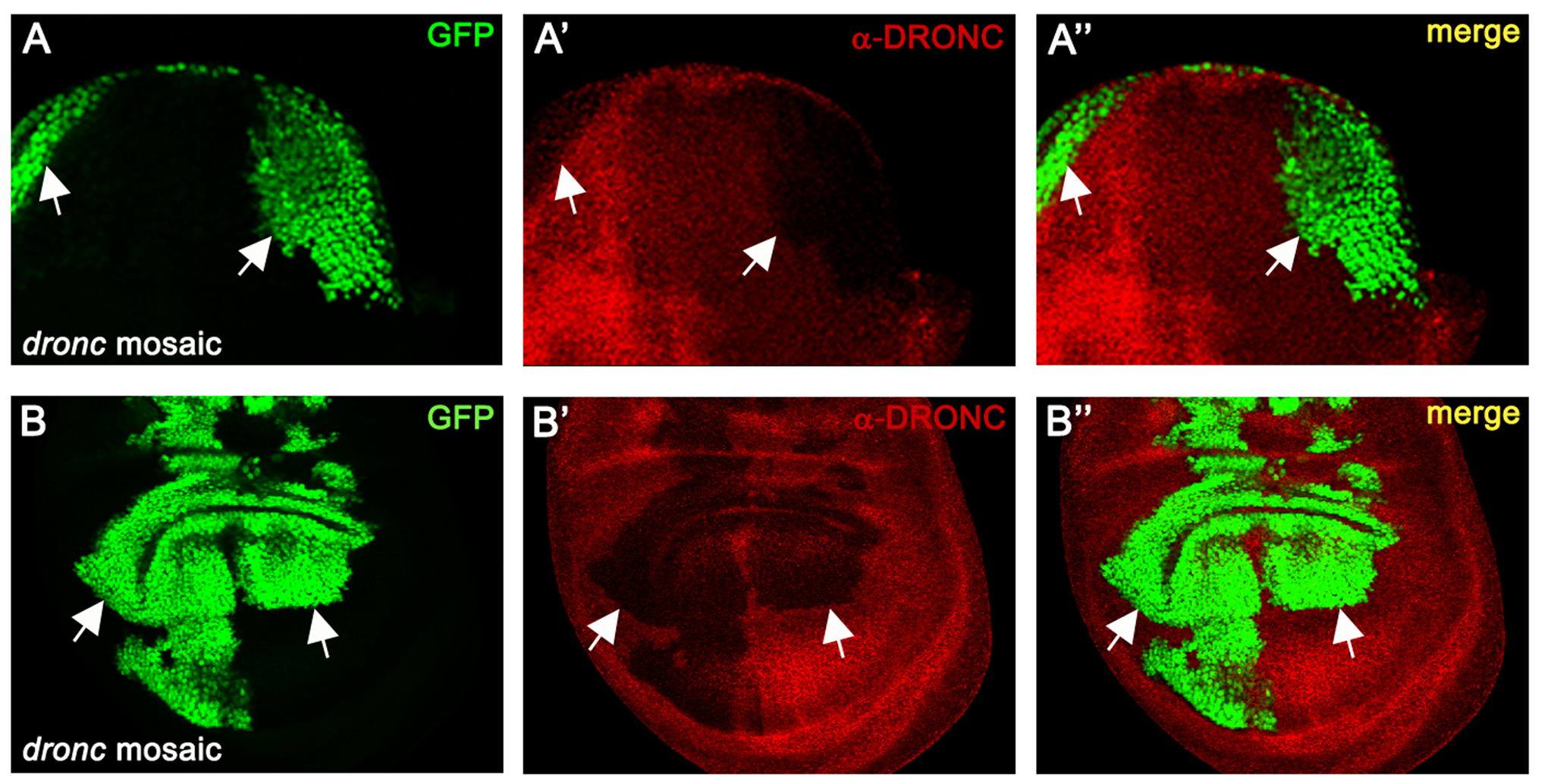

Supplement: Figure S3 — Specificity of the anti-DRONC antibody. The specificity of the anti-DRONC antibody used for immunofluorescence in Figure 3, Figure 4, and Figure 5 was verified in droncI29 mosaic eye (A) and wing (B) imaginal discs. The droncI29 allele contains a premature STOP codon at position 53 [11]. droncI29 clones were induced using the MARCM system, hence they are positively marked by GFP (arrows). The anti-DRONC antibody does not produce labeling signals in the mutant clones (arrows in A′ and B′, and the merge in A″ and B″), demonstrating that it is specific for DRONC. Genotype: hs-FLP; droncI29 FRT80/ubi-GFP FRT80. (TIF) [file pgen.1002261.s003.tif]

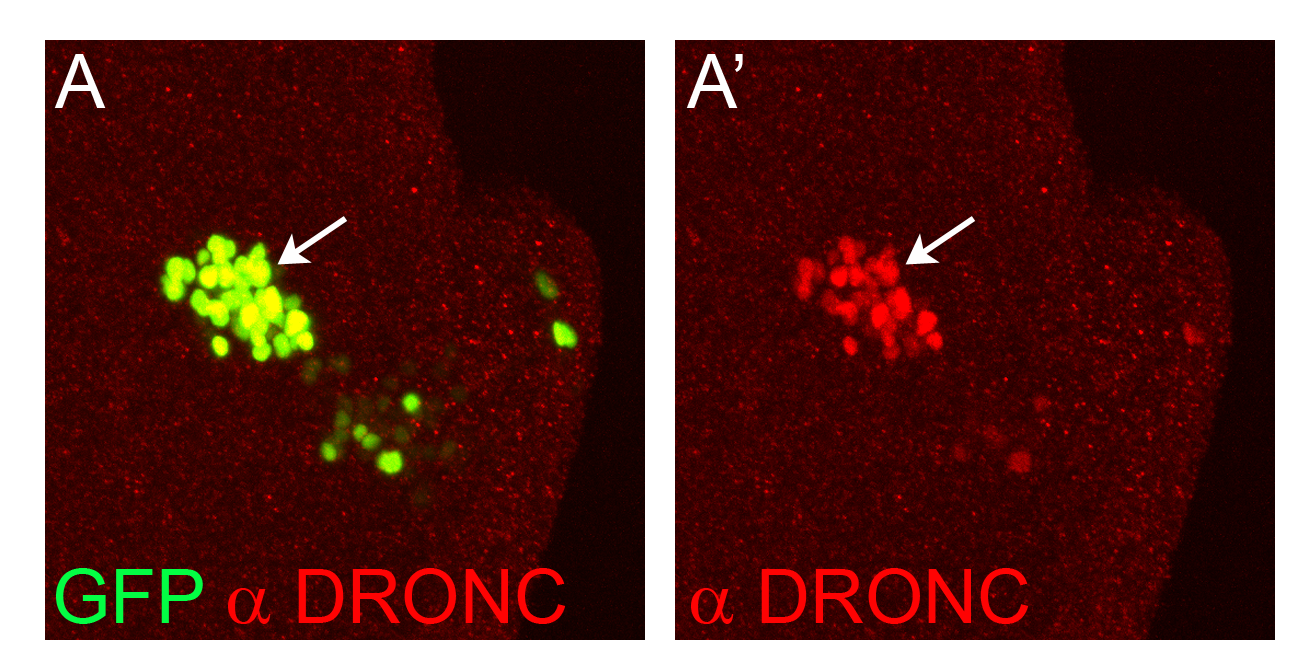

Supplement: Figure S4 — “Undead” diap122-8s cells accumulate DRONC protein autonomously. (A, A′) Using MARCM, p35-expressing, ‘undead’ diap122-8s mutant clones (green) were induced in eye discs and labeled for DRONC protein (red). DRONC protein autonomously accumulates in P35-expressing diap122-8s clones (arrows). Similar results were obtained in wing discs (data not shown). Genotype: hs-FLP tub-GAL4 UAS-GFP/+; UAS-p35/+; diap122-8s FRT80/tub-GAL80 FRT80. (TIF) [file pgen.1002261.s004.tif]
